# *N*-[Bis(dimethyl­amino)phosphinoyl]-2,2,2-trichloro­acetamide

**DOI:** 10.1107/S1600536810013589

**Published:** 2010-04-17

**Authors:** Oleksiy V. Amirkhanov, Olesia V. Moroz, Kateryna O. Znovjyak, Elizaveta A. Trush, Tetyana Yu. Sliva

**Affiliations:** a Kyiv National Taras Shevchenko University, Department of Chemistry, Volodymyrska str. 64, 01601 Kyiv, Ukraine

## Abstract

In the title compound, C_6_H_13_Cl_3_N_3_O_2_P or CCl_3_C(O)NHP(O)(N(CH_3_)_2_), the phosphinoyl group is synclinal to the carbonyl group and acts as an acceptor for an inter­molecular N—H⋯O hydrogen bond from the amide group as the donor.

## Related literature

For the biological and pharmacological properties of carbacyl­amido­phosphate derivatives, see: Adams *et al.* (2002 ▶); Grimes *et al.* (2008 ▶). For structural and conformation studies of related mol­ecules, see: Gholivand *et al.* (2008*a* ▶,*b* ▶); Skopenko *et al.* (2004 ▶); Znovjyak *et al.* (2009*a* ▶,*b* ▶). For details of the synthesis, see: Kirsanov & Derkach (1956 ▶). For P—O bond lengths in compounds with amide substituents close to phospho­rus atoms, see: Rebrova *et al.* (1982 ▶).
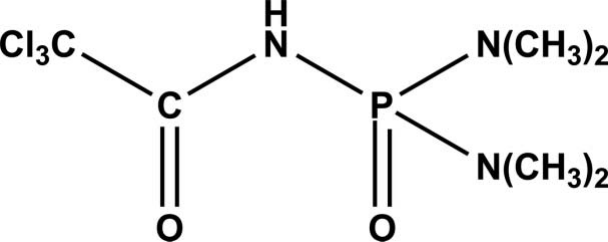


## Experimental

### 

#### Crystal data

C_6_H_13_Cl_3_N_3_O_2_P
*M* *_r_* = 296.51
Orthorhombic, 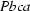

*a* = 15.794 (3) Å
*b* = 15.820 (3) Å
*c* = 9.739 (2) Å
*V* = 2433.4 (8) Å^3^
*Z* = 8
Mo *K*α radiation
μ = 0.87 mm^−1^
*T* = 100 K
0.60 × 0.40 × 0.20 mm

#### Data collection

Oxford Diffraction Xcalibur Sapphire2 (large Be window) diffractometer
Absorption correction: Gaussian (Coppens *et al.*, 1965 ▶) *T*_min_ = 0.624, *T*_max_ = 0.845
35140 measured reflections
5631 independent reflections
4869 reflections with *I* > 2σ(*I*)
*R*_int_ = 0.032

#### Refinement

*R*[*F* ^2^ > 2σ(*F* ^2^)] = 0.030
*wR*(*F* ^2^) = 0.072
*S* = 1.15
5631 reflections
144 parameters
H atoms treated by a mixture of independent and constrained refinement
Δρ_max_ = 0.46 e Å^−3^
Δρ_min_ = −0.40 e Å^−3^

### 

Data collection: *CrysAlis CCD* (Oxford Diffraction, 2006 ▶); cell refinement: *CrysAlis RED* (Oxford Diffraction, 2006 ▶); data reduction: *CrysAlis RED*; program(s) used to solve structure: *SHELXS97* (Sheldrick, 2008 ▶); program(s) used to refine structure: *SHELXL97* (Sheldrick, 2008 ▶); molecular graphics: *ORTEP-3 for Windows* (Farrugia, 1997 ▶); software used to prepare material for publication: *WinGX* (Farrugia, 1999 ▶).

## Supplementary Material

Crystal structure: contains datablocks I, global. DOI: 10.1107/S1600536810013589/jh2144sup1.cif

Structure factors: contains datablocks I. DOI: 10.1107/S1600536810013589/jh2144Isup2.hkl

Additional supplementary materials:  crystallographic information; 3D view; checkCIF report

## Figures and Tables

**Table 1:** Hydrogen-bond geometry (Å, °)

*D*—H⋯*A*	*D*—H	H⋯*A*	*D*⋯*A*	*D*—H⋯*A*
N1—H1*N*⋯O1^i^	0.828 (16)	1.968 (16)	2.7586 (11)	159.3 (16)

Symmetry code: (i) 
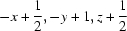
.

**Fig. 1.**
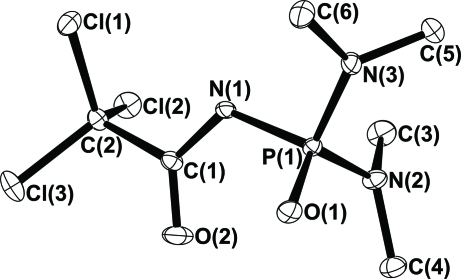
Structural representation of 1 with atom numbering scheme and 50% probability thermal ellipsoid. The hydrogen atoms are omitted for clarity.

**Fig. 2.**
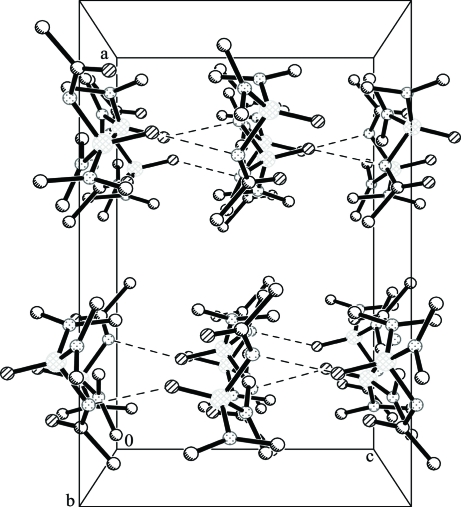
A projection of packing diagram of the title compound along the y direction. Hydrogen bonds are indicated by dashed lines. H and Cl atoms are omitted for clarity.

## supplementary crystallographic information

### Comment

P, N-substituted analogues of β-diketones – carbacylamidophosphates (CAPh), which comprise C(O)NHP(O) structural fragment, have attracted attention because of their using in pharmacology, as insecticides, pesticides and ureas inhibitor (Adams *et al.*, 2002; Grimes *et al.*, 2008). A variety of new coordination compounds with s-, *p*-, d- and f-metals based on the CAPh have been synthesized and characterized up to date (Skopenko *et al.*, 2004; Znovjyak *et al.*, 2009a). Thus, the syntheses and structure analysis of CAPh have been of increased interest (Gholivand *et al.*, 2008a). In this paper we report the crystal structure of a new CAPh – *N*-[bis(dimethylamino)phosphinoyl]-2,2,2-trichloroacetamide (1).

In the crystal packing molecules of 1 are linked to each other by intermolecular hydrogen bonds (N–H···O), where amide nitrogen atom of one molecule acts as donor and oxygen atom of phosphinoyl group of neighboring molecule acts as acceptor (Fig. 2). The bond distance P(1)—O(1) (1.478 (1) Å) is typical for compounds with amide substituents close to phosphorus atoms (Rebrova *et al.*, 1982) and CAPh (Znovjyak *et al.*, 2009b). The values of C(1)—O(2) and C(1)—N(1) are 1.215 (1) Å and 1.355 (1) Å, respectively and close to the corresponding values of the CAPh (Gholivand *et al.*, 2008b). The P(1)—N(1) (1.714 (1) Å) distance in 1 is longer on average by 0.08 Å than P—N bond distances between amide substituents and phosphorus atoms (P(1)—N(2), P(1)—N(3)). The geometry around the phosphorus atom in 1 can be described as a distorted tetrahedron and which is similar to early reported CAPh. The O(1)—P(1)—N(1) angle value is close to near tetrahedral one (109.61 (4)°), while other O—P—N angels have values 113.19 (4)° and 116.53 (4)°, that may be explained by the repulsion between amide substituents and PO group. Fragment O(2)—C(1)—N(1)—P(1) is close to planarity (the torsion angle is -176.6°).

### Experimental

The dichloranhydride of trichloroacetylamidophosphoric acid (CCl_3_C(O)NHP(O)Cl_2_) was prepared according to the method reported by Kirsanov (Kirsanov *et al.*, 1956).

The dioxane solution (200 ml) of CCl_3_C(O)NHP(O)Cl_2_ (27.9 g, 0.1 mol) was placed in a three-neck round-bottomed flask and cooled by ice to 268 K. Then the dry dimethylamine (18.03 g, 0.4 mol) was bubbled through the dioxane solution of CCl_3_C(O)NHP(O)Cl_2_ under stirring until the solution became alkaline. The temperature was not allowed to rise above 278 K. The stirring was continued for 1 h and the solution was left under ambient conditions. HN(CH_3_)_2_^.^HCl was filtered off after 12 h and the filtrate was evaporated. The oily precipitate of 1 was isolated and recrystallized from 2-propanol which led to formation of white crystalline powder (yield 80%). White needle-shaped crystals suitable for X-ray analysis were formed over a period of 5 days from the 2-propanol/hexane solution. IR (KBr pellet, cm^-1^): 3070 (ν(NH)), 1715 (ν(CO)), 1205 (ν(PO)), 1000 (ν(PN_amine_)), 867 (ν(PN_amide_)), 676 (ν(CCl)).

### Refinement

Refinement of F^2^ against ALL reflections. The weighted R-factor wR and goodness of fit S are based on F^2^, conventional R-factors R are based on F, with F set to zero for negative F^2^. The threshold expression of F^2^ > 2σ(F^2^) is used only for calculating R-factors(gt) etc. and is not relevant to the choice of reflections for refinement. R-factors based on F^2^ are statistically about twice as large as those based on F, and R- factors based on ALL data will be even larger.

### Crystal data

**Table:**

C_6_H_13_Cl_3_N_3_O_2_P	*F*(000) = 1216
*M**_r_* = 296.51	*D*_x_ = 1.619 Mg m^−^^3^
Orthorhombic, *P**b**c**a*	Mo *K*α radiation, λ = 0.71073 Å
Hall symbol: -P 2ac 2ab	Cell parameters from 35140 reflections
*a* = 15.794 (3) Å	θ = 3.6–36.1°
*b* = 15.820 (3) Å	µ = 0.87 mm^−^^1^
*c* = 9.739 (2) Å	*T* = 100 K
*V* = 2433.4 (8) Å^3^	Block, white
*Z* = 8	0.60 × 0.40 × 0.20 mm

### Data collection

**Table:**

Oxford Diffraction Xcalibur Sapphire2 (large Be window) diffractometer	5631 independent reflections
Radiation source: Enhance (Mo) X-ray Source	4869 reflections with *I* > 2σ(*I*)
graphite	*R*_int_ = 0.032
Detector resolution: 8.3359 pixels mm^-1^	θ_max_ = 36.1°, θ_min_ = 3.6°
ω scans	*h* = −26→26
Absorption correction: gaussian Coppens et al. (1965)	*k* = −25→25
*T*_min_ = 0.624, *T*_max_ = 0.845	*l* = −12→15
35140 measured reflections	

### Refinement

**Table:**

Refinement on *F*^2^	Primary atom site location: structure-invariant direct methods
Least-squares matrix: full	Secondary atom site location: difference Fourier map
*R*[*F*^2^ > 2σ(*F*^2^)] = 0.030	Hydrogen site location: inferred from neighbouring sites
*wR*(*F*^2^) = 0.072	H atoms treated by a mixture of independent and constrained refinement
*S* = 1.15	*w* = 1/[σ^2^(*F*_o_^2^) + (0.0336*P*)^2^ + 0.5268*P*] where *P* = (*F*_o_^2^ + 2*F*_c_^2^)/3
5631 reflections	(Δ/σ)_max_ = 0.001
144 parameters	Δρ_max_ = 0.46 e Å^−^^3^
0 restraints	Δρ_min_ = −0.40 e Å^−^^3^

### Special details

**Table:**

Geometry. All s.u.'s (except the s.u. in the dihedral angle between two l.s. planes) are estimated using the full covariance matrix. The cell s.u.'s are taken into account individually in the estimation of s.u.'s in distances, angles and torsion angles; correlations between s.u.'s in cell parameters are only used when they are defined by crystal symmetry. An approximate (isotropic) treatment of cell s.u.'s is used for estimating s.u.'s involving l.s. planes.

### Fractional atomic coordinates and isotropic or equivalent isotropic displacement parameters (Å^2^)

**Table:**

	*x*	*y*	*z*	*U*_iso_*/*U*_eq_	
P1	0.285320 (15)	0.522358 (14)	0.42665 (2)	0.00982 (5)	
C1	0.14518 (6)	0.60952 (6)	0.48883 (9)	0.01181 (14)	
C2	0.06928 (6)	0.62610 (5)	0.58869 (9)	0.01177 (15)	
Cl1	0.033556 (15)	0.534225 (14)	0.67522 (3)	0.01615 (5)	
Cl2	0.104133 (16)	0.701650 (14)	0.71094 (2)	0.01672 (5)	
Cl3	−0.015884 (16)	0.668869 (15)	0.49346 (3)	0.01985 (6)	
O1	0.26191 (5)	0.51406 (5)	0.28033 (7)	0.01480 (12)	
O2	0.15469 (5)	0.65692 (5)	0.39188 (8)	0.02050 (15)	
N1	0.19672 (5)	0.54418 (5)	0.52192 (8)	0.01086 (13)	
N2	0.35444 (5)	0.59761 (5)	0.45503 (8)	0.01341 (14)	
N3	0.32000 (5)	0.43758 (5)	0.50197 (9)	0.01413 (14)	
C3	0.36307 (7)	0.63290 (7)	0.59326 (10)	0.01877 (18)	
H3A	0.3449	0.5908	0.6610	0.028*	
H3B	0.3277	0.6835	0.6014	0.028*	
H3C	0.4224	0.6479	0.6099	0.028*	
C4	0.37770 (7)	0.65551 (6)	0.34435 (11)	0.01709 (17)	
H4A	0.3434	0.7070	0.3510	0.026*	
H4B	0.3675	0.6282	0.2556	0.026*	
H4C	0.4378	0.6702	0.3523	0.026*	
C5	0.40306 (6)	0.42489 (7)	0.56243 (11)	0.01898 (18)	
H5A	0.4371	0.4762	0.5507	0.028*	
H5B	0.4313	0.3774	0.5169	0.028*	
H5C	0.3969	0.4126	0.6605	0.028*	
C6	0.26438 (7)	0.36368 (6)	0.51134 (12)	0.01950 (19)	
H6A	0.2906	0.3158	0.4636	0.029*	
H6B	0.2098	0.3767	0.4686	0.029*	
H6C	0.2556	0.3491	0.6081	0.029*	
H1N	0.1961 (10)	0.5241 (9)	0.6005 (17)	0.023 (4)*	

### Atomic displacement parameters (Å^2^)

**Table:**

	*U*^11^	*U*^22^	*U*^33^	*U*^12^	*U*^13^	*U*^23^
P1	0.01062 (9)	0.01090 (9)	0.00794 (9)	0.00005 (7)	0.00106 (7)	−0.00067 (7)
C1	0.0138 (4)	0.0121 (3)	0.0095 (3)	0.0019 (3)	−0.0007 (3)	−0.0001 (3)
C2	0.0123 (3)	0.0113 (3)	0.0117 (4)	0.0021 (3)	−0.0013 (3)	−0.0018 (3)
Cl1	0.01546 (10)	0.01469 (9)	0.01831 (11)	−0.00068 (7)	0.00464 (8)	0.00048 (7)
Cl2	0.02070 (10)	0.01642 (10)	0.01304 (10)	−0.00216 (7)	−0.00055 (8)	−0.00503 (7)
Cl3	0.01663 (10)	0.01936 (10)	0.02357 (12)	0.00705 (8)	−0.00704 (9)	−0.00265 (8)
O1	0.0174 (3)	0.0188 (3)	0.0082 (3)	−0.0010 (2)	0.0010 (2)	−0.0026 (2)
O2	0.0275 (4)	0.0196 (3)	0.0144 (3)	0.0069 (3)	0.0044 (3)	0.0073 (3)
N1	0.0119 (3)	0.0131 (3)	0.0076 (3)	0.0026 (2)	0.0013 (2)	0.0018 (2)
N2	0.0165 (3)	0.0145 (3)	0.0092 (3)	−0.0034 (3)	0.0001 (3)	−0.0002 (2)
N3	0.0116 (3)	0.0120 (3)	0.0188 (4)	0.0006 (2)	−0.0001 (3)	0.0027 (3)
C3	0.0226 (5)	0.0203 (4)	0.0134 (4)	−0.0056 (3)	−0.0021 (3)	−0.0032 (3)
C4	0.0195 (4)	0.0157 (4)	0.0160 (4)	−0.0041 (3)	0.0007 (3)	0.0031 (3)
C5	0.0147 (4)	0.0214 (4)	0.0208 (5)	0.0021 (3)	−0.0032 (4)	0.0047 (4)
C6	0.0177 (4)	0.0124 (4)	0.0284 (5)	−0.0003 (3)	0.0031 (4)	0.0013 (3)

### Geometric parameters (Å, °)

**Table:**

P1—O1	1.4780 (8)	N3—C6	1.4652 (13)
P1—N3	1.6239 (8)	C3—H3A	0.9800
P1—N2	1.6388 (8)	C3—H3B	0.9800
P1—N1	1.7141 (8)	C3—H3C	0.9800
C1—O2	1.2151 (11)	C4—H4A	0.9800
C1—N1	1.3546 (12)	C4—H4B	0.9800
C1—C2	1.5658 (13)	C4—H4C	0.9800
C2—Cl3	1.7684 (9)	C5—H5A	0.9800
C2—Cl1	1.7723 (10)	C5—H5B	0.9800
C2—Cl2	1.7745 (9)	C5—H5C	0.9800
N1—H1N	0.828 (16)	C6—H6A	0.9800
N2—C4	1.4614 (13)	C6—H6B	0.9800
N2—C3	1.4637 (13)	C6—H6C	0.9800
N3—C5	1.4518 (13)		
			
O1—P1—N3	116.53 (4)	N2—C3—H3A	109.5
O1—P1—N2	113.19 (4)	N2—C3—H3B	109.5
N3—P1—N2	107.40 (4)	H3A—C3—H3B	109.5
O1—P1—N1	109.61 (4)	N2—C3—H3C	109.5
N3—P1—N1	101.37 (4)	H3A—C3—H3C	109.5
N2—P1—N1	107.83 (4)	H3B—C3—H3C	109.5
O2—C1—N1	125.55 (9)	N2—C4—H4A	109.5
O2—C1—C2	118.29 (8)	N2—C4—H4B	109.5
N1—C1—C2	116.12 (8)	H4A—C4—H4B	109.5
C1—C2—Cl3	108.70 (6)	N2—C4—H4C	109.5
C1—C2—Cl1	113.68 (6)	H4A—C4—H4C	109.5
Cl3—C2—Cl1	108.72 (5)	H4B—C4—H4C	109.5
C1—C2—Cl2	106.98 (6)	N3—C5—H5A	109.5
Cl3—C2—Cl2	109.28 (5)	N3—C5—H5B	109.5
Cl1—C2—Cl2	109.40 (5)	H5A—C5—H5B	109.5
C1—N1—P1	121.03 (7)	N3—C5—H5C	109.5
C1—N1—H1N	120.3 (11)	H5A—C5—H5C	109.5
P1—N1—H1N	115.7 (11)	H5B—C5—H5C	109.5
C4—N2—C3	114.58 (8)	N3—C6—H6A	109.5
C4—N2—P1	119.89 (7)	N3—C6—H6B	109.5
C3—N2—P1	119.62 (7)	H6A—C6—H6B	109.5
C5—N3—C6	113.97 (8)	N3—C6—H6C	109.5
C5—N3—P1	127.02 (7)	H6A—C6—H6C	109.5
C6—N3—P1	119.00 (7)	H6B—C6—H6C	109.5
			
O2—C1—C2—Cl3	32.74 (11)	N3—P1—N2—C4	138.23 (8)
N1—C1—C2—Cl3	−149.48 (7)	N1—P1—N2—C4	−113.24 (8)
O2—C1—C2—Cl1	153.97 (8)	O1—P1—N2—C3	159.66 (7)
N1—C1—C2—Cl1	−28.25 (10)	N3—P1—N2—C3	−70.29 (8)
O2—C1—C2—Cl2	−85.16 (10)	N1—P1—N2—C3	38.24 (9)
N1—C1—C2—Cl2	92.62 (8)	O1—P1—N3—C5	118.05 (9)
O2—C1—N1—P1	1.02 (14)	N2—P1—N3—C5	−10.10 (10)
C2—C1—N1—P1	−176.58 (6)	N1—P1—N3—C5	−123.08 (9)
O1—P1—N1—C1	−53.07 (8)	O1—P1—N3—C6	−61.17 (9)
N3—P1—N1—C1	−176.80 (7)	N2—P1—N3—C6	170.68 (7)
N2—P1—N1—C1	70.54 (8)	N1—P1—N3—C6	57.70 (8)
O1—P1—N2—C4	8.18 (9)		

### Hydrogen-bond geometry (Å, °)

**Table:**

*D*—H···*A*	*D*—H	H···*A*	*D*···*A*	*D*—H···*A*
N1—H1N···O1^i^	0.828 (16)	1.968 (16)	2.7586 (11)	159.3 (16)

Symmetry codes: (i) −*x*+1/2, −*y*+1, *z*+1/2.
